# Parents’ Reported Experiences When Having a Child with Cataract—Important Aspects of Self-Management Obtained from the Pediatric Cataract Register (PECARE)

**DOI:** 10.3390/ijerph17176329

**Published:** 2020-08-31

**Authors:** Jenny Gyllén, Gunilla Magnusson, Anna Forsberg

**Affiliations:** 1 Department of Clinical Neuroscience, Institute of Neuroscience and Physiology, Sahlgrenska Academy, University of Gothenburg, 40530 Gothenburg, Sweden; gunilla.magnusson@vgregion.se; 2 Region Västra Götaland, Department of Ophthalmology, Sahlgrenska University Hospital, 41345 Mölndal, Sweden; 3 Research Group: Care in High Tech Environments, Institute of Health Sciences, Lund University, 22100 Lund, Sweden; anna.forsberg@med.lu.se; 4 Thoracic Unit, Skåne University Hospital, 22185 Lund, Sweden

**Keywords:** pediatric cataract, fatigue, parents, family centered care

## Abstract

Parents are a crucial part in the care of children with pediatric cataract. The aim of this study was to explore and explain sense of coherence, family self-efficacy, perceived social support, fatigue and parent reported experiences (PREM), in order to improve clinical care. Questionnaires were sent to the parents of children registered in the Swedish national Pediatric Cataract Register, PECARE, from 2006–2019 (*n* = 506). The response rate was 46% (*n* = 231), 185 mothers and 44 fathers with a mean age of 40.39 years (SD ± 6.41 years). In total, 38% of the parents reported severe fatigue, and mothers were more burdened than fathers. Sense of coherence was strongly related to fatigue, especially among parents of children with bilateral cataract. Mental fatigue and reduced motivation explained 45% of the variation in sense of coherence. Being taken seriously by the ophthalmological clinic explained over 60% of the variation in satisfaction with care when controlled for parents’ age and gender. In conclusion, fatigue is important to take in consideration when interacting with parents of children with cataract, especially those with bilateral cataract. Being taken seriously is the key marker of satisfaction with care and support from professionals. In addition to fatigue, the parents’ age and life situation affect how they perceive their own, as well as the professionals’ effort, and should be considered when tailoring family-centered care.

## 1. Introduction

Pediatric cataract is a rare condition. It can be classified using age of onset, etiology and morphology. In Sweden, about 40 children are born each year with congenital/infantile cataract, representing an occurrence of 36 per 100,000 births [1]. Children under the age of three years are operated on at two hospitals in Sweden, appointed by the Swedish National Board of Health and Welfare to perform National Specialized Medical Care of children with pediatric cataract. In Sweden, all children under the age of eight years undergoing surgery for cataract are registered in the Pediatric Cataract Register (PECARE), a national quality register, with the aim of optimizing screening strategies and more effective treatment of pediatric cataract. The goal is to constitute a national base for quality assurance and to define and analyze unexpected treatment outcomes. Since its launch in 2006, the register has gathered data on 731 children (1029 eyes) (1 June 2020) [2]. The PECARE enables us to investigate a geographically based national cohort of children and their parents, giving us a unique opportunity to grasp the parents’ perspective on the care provided, their own efforts as care givers, their self-management and the challenges experienced.

This study is based on the assumption that parents who experience well-being and self-efficacy can care for and support their child with adequate self-management support from professionals. Two previous inductive studies show that the uncertainty that can occur among parents can become a barrier to self-efficacy and that parents balance their child’s inability and ability through a process comprising four main categories; mastering, collaborating, facilitating and adapting [3,4]. It is important to explore a larger group of parents determine whether the same patterns are present, which constitutes the rationale for this study.

What we do know is that being a parent of a child with pediatric cataract is demanding. Parents struggle to maintain a balance between their child’s ability and inability [3,4,5,6]. The journey starts at the time of the diagnosis, usually at birth, and continues for years, with numerous visits to the eye clinic for check-ups. The parents are expected to be alert to signs of complications, such as infections and glaucoma. Setbacks such as surgery for glaucoma and visual axis opacification, loss of visual acuity and lack of support throw parents back into uncertainty. Studies reveal that adherence to treatment such as handling contact lenses, eye drops and patching and devotion to stimulating their child can be very challenging [3,4,7,8,9,10,11]. This challenge requires an ophthalmological team that is engaged and works in collaboration with the parents, to ensure the best possible visual outcome for the children.

Self-management reflects the child’s and caregiver’s active engagement in knowing about the disease, recognizing signs and symptoms, as well as decision-making abilities enabling them to seek help, in order to prevent potential complications [12]. Self-management is linked to self-efficacy, which in this context is described as parents’ belief in their ability to perform the parenting role [13]. A possible barrier to self-management is fatigue, described as an overwhelming feeling of physical and mental exhaustion not easily relieved by rest [14,15].

A key clinical question is how to organize the care of families in which a child has pediatric cataract to provide adequate and relevant self-management support. The Innovation for Chronic Conditions (ICCC) framework presented by the World Health Organization (WHO) proposes that forming partnerships between informed, motivated and prepared patients and families, a motivated healthcare team and informed community partners constitutes the basis for better outcomes in those who are chronically ill [16]. Healthcare should promote continuity and coordination, encourage quality through leadership and incentives, organize and equip healthcare teams, make use of information systems and support self-management [16]. Healthcare systems organized with focus on self-care and chronic illness management report better long-term outcomes in chronically ill populations [17,18]. Self-efficacy, which is possible to enhance, has an important effect on self-care behavior, and constitutes the foundation of self-management programs [19].

A previous study concluded that parents’ main concerns were mastering the child’s disability and ability through a process comprising four main categories; mastering, collaborating, facilitating and adapting [3]. As there is a lack of scientific knowledge regarding the life situation of parents of children with cataract, the aim of this study was to explore and explain sense of coherence, family self-efficacy, perceived social support, fatigue and parent reported experiences of caring for a child with pediatric cataract, in order to improve clinical care.

## 2. Methods

### 2.1. Participants and Selection

All parents of children registered in the PECARE since 2006 (*n* = 506 at the start of the study in March 2019) were included. Exclusion criteria were cataract caused by juvenile idiopathic arthritis (JIA), lens luxation and trauma. Demographic data retrieved from the parents included information about age, gender, ethnic background, education, occupation, social status and whether or not the parent was the caretaker of the child. One question dealt with whether the parent considered sharing the work of caring for the child with cataract with the other parent. No data were missing from the PECARE regarding the children’s age, gender, diagnoses, laterality and BCVA, as they are mandatory fields in the computerized reporting system. The PECARE has 95% coverage (from January 2007–December 2018).

### 2.2. Instruments

Four instruments were selected to cover aspects of being a parent of a child with cataract:

1. Sense of coherence (SOC) describes a person’s ability to cope with inner and outer stressors operationalized to comprehensibility, manageability and meaningfulness [20]. A person with a strong SOC will view stressful stimuli as predictable, understandable (comprehensible) and as challenges that are worthy of engaging in (meaningful), as well as believing that she/he possesses or can acquire the resources to overcome stressful stimuli (manageable) [21]. We used the short form of the SOC comprising 13-items on a 7-point Likert scale with a score range of 13–191, which has been validated for Swedish use by [22].
2. Fatigue was explored by the Multidimensional Fatigue Inventory Scale (MFI-19) [23], which consists of 20 questions (one question was omitted in the present study) with five subscales: general fatigue (GF), physical fatigue (PhF), reduced activity (RA), reduced motivation (RM) and mental fatigue (MF). The MFI-19 has been validated for Swedish use by [24].
3. Parental social support was measured by the Multidimensional scale of perceived social support (MSPSS) [25]. Social support is composed of emotional and instrumental support, where the latter includes providing tangible help, such as child care support, transportation, household assistance and providing money or shelter [26]. The MSPSS scale addresses the subjective assessment of social support, and is designed to evaluate perceptions from three specific sources: family, friends and significant other [25]. It consists of 12 questions and has been validated for Swedish use by [27].
4. The Perceived Collective Family Efficacy Scale (PFES) was used to explore self-efficacy, which is defined as the patient’s confidence in her/his ability to adequately manage the demands of her/his illness [28]. The PFES contains 19 items assessing belief in the family’s efficacy to operate as a whole system in accomplishing tasks necessary for family functioning [29].

Additionally, there were four patient reported experience measures (PREMs), where the parents were asked to respond to four questions on a scale ranging from 1–100.

- How satisfied are you with the treatment of your child’s cataract?
- How satisfied are you with your efforts as a parent of your child with a cataract?
- Do you experience that you receive enough support from the clinic that treats your child’s cataract?
- Do you feel that you have been taken seriously by the clinic that treats your child’s cataract?

A letter with information about the study and an invitation to participate was sent to all parents whose children met the inclusion criteria and were registered in the PECARE (*n* = 506). When the informed consent form was returned, the questionnaires were sent out. If the questionnaires were not returned, two reminders were sent, which resulted in 231 returned questionnaires. The questionnaires included open-ended questions and 30% of the parents responded. These will be analyzed and presented in a separate paper.

### 2.3. Statistics and Ethics

The SPSS for Windows version 25.0 (IBM Corp., Armonk, NY, USA) was used when analyzing data, which were mainly ordinal. Descriptive statistics (parental demographics, socio-demographics etc.) were presented with frequencies. We chose to dichotomize parents’ age, education, social status, occupation and native country, and used an established cut off for general fatigue. For the children, we dichotomized age at the time of the study and age at surgery. When testing for differences between two un-paired groups, we applied the Mann–Whitney U Test. In order to explore relationships between the different phenomena, we employed Spearman’s Rho. Hierarchical multiple regression was used to assess explanatory factors of family self-efficacy and satisfaction with care, after controlling for the influence of age and gender. Differences with a *p*-value ≤ 0.05 were considered significant.

The study was conducted in accordance with the principles of the Declaration of Helsinki and the Medical Research Involving Human Subjects Act. Approval was obtained from the Swedish Ethical Review Authority (20 19-00836/1234-18).

## 3. Results

### 3.1. Findings in the Whole Group

The response rate was 46% (*n* = 231). The respondents were predominantly mothers (80%) from Sweden (81.4%) with a university education (69.7%), who were working or studying (87%). They were living in a conjugal family (87%) and had a large support network, i.e., extensive positive resources for taking care of their child.

All parents who responded reported being the caretaker of their child with cataract and 87% (*n* = 201) stated that they shared the work with the other parent. Furthermore, 52.5% (*n* = 121) of the children were operated on within their first year. Demographics and clinical characteristics of parents and children are presented in Table 1 Measurements of self-efficacy and experiences of care for the whole group are presented in Table 2 In addition, 38% of the parents scored >12 in GF, meaning that they were severely fatigued.

The nationality of the parents, the child’s age, or if the child had complications/re-operations did not correlate to any other variables in this study.

### 3.2. Subgroup Analysis

#### 3.2.1. Parents of Children Suffering from Bilateral Cataract and Co-Existing Systemic Disorders

Co-existing systemic disorders were predominantly found in children with bilateral cataract (89%). Parents of a child with bilateral cataract reported significantly more fatigue on four subscales of fatigue than those parenting children with unilateral cataract GF (*p* = 0.007), PhF (*p* = 0.030), RM (*p* = 0.005) and MF (*p* = 0.019). However, these parents were more satisfied with the treatment (*p* = 0.009), their own efforts (*p* = 0.004), support from the ophthalmological clinic (*p* = 0.000), and felt that they were taken seriously (*p* = 0.050) compared to parents of children with a unilateral cataract.

If the child had co-existing systemic disorders, the parents reported lower family self-efficacy (*p* = 0.029) and lower support from friends (*p* = 0.049) than if their child only had cataract. Parents of boys were more satisfied with their own efforts as a parent (*p* = 0.03) compared to parents of girls.

#### 3.2.2. Parental Gender Differences

Bearing in mind that mothers constituted 80% of the respondents, they reported more GF (*p* = 0.028) and PhF (*p* = 0.052) than fathers. Furthermore, fathers reported less meaningfulness (*p* < 0.019) and lower social support from friends (*p* = 0.010).

#### 3.2.3. Parents’ Ages and Life Situation

Parents who were not living with the other parent reported more difficulties managing the situation (*p* = 0.049), and experienced less support from the ophthalmological clinic (*p* = 0.024) compared to parents living together. Those parents not sharing the work with the other parent (9.5%) reported a lower SOC in the manageability (*p* = 0.038) and meaningfulness (*p* = 0.056) subscales. They also expressed more GF (*p* = 0.003), PhF (*p* = 0.002), RA (*p* = 0.011) and MF (*p* = 0.015). Additionally, these parents reported lower support from their significant other (*p* = 0.036) and family (*p* = 0.017), as well as lower family self-efficacy (*p* = 0.023).

Compared with parents younger than 40 years, those older than 40 years reported less motivation due to fatigue (*p* = 0.041), and were less satisfied with the treatment their child received (*p* = 0.052). They also reported lower support from their significant other (*p* = 0.008). Parents with a university or college education expressed less satisfaction with their own efforts in caring for their child with cataract (*p* = 0.049) than those with a mandatory education. Parents on parental leave reported reduced activity (*p* = 0.014) compared to those working.

### 3.3. Relationships

There was a strong relationship between the overall SOC total and PhF (r_s_ = 0.516), RA (r_s_ = 0.510), RM (r_s_ = 0.508) and MF (r_s_ = 0.535). We therefore used linear multiple regression when testing the fatigue dimensions as independent variables, possibly explaining the variation in total SOC. Only RM (Beta = 0.239, *p* ≤ 0.0001) and MF (Beta = 0.236, *p* ≤ 0.0001) contributed significantly to the variance in total SOC. Thus, we proceeded with hierarchical multiple regression to assess the ability of RM and MF, to predict levels of total SOC after controlling for the influence of parents’ gender and age. Preliminary analyses were conducted to ensure no violation of the assumptions of normality, linearity, multicollinearity and homoscedasticity. Age and gender were entered at step 1, explaining 12.6% of the variance in the total SOC. After the entry of RM and MF at step 2, the total variance explained by the model was 44.9%, *p* = ≤ 0.0001. RM and MF explained an additional 32.3% of the variance in the total SOC, *R*-squared change = 0.323, *F* change (2,224) = 65.65, *p* ≤ 0.0001. In the final model, MF recorded a slightly higher beta value (*beta* = −0.34, *p* ≤ 0.0001) than RM (*beta* = −0.33, *p* ≤ 0.0001).

#### 3.3.1. Parents’ Reported Experience Measures

Satisfaction with the treatment provided by the ophthalmological clinic was moderately related to satisfaction with one’s own effort as a parent (r_s_ = 0.456), and strongly related to support from the clinic (r_s_ = 0.673) and being taken seriously (r_s_ = 0.507). Satisfaction with one’s own effort was related to support from the clinic (r_s_ = 0.447) and weakly related to being taken seriously (r_s_ = 0.256). Finally, support from the clinic was strongly related to the sense of being taken seriously (r_s_ = 0.718). Thus, we proceeded with hierarchical multiple regression to assess the ability of being taken seriously to predict experienced levels of support from the clinic, after controlling for the influence of parents’ gender and age. Preliminary analyses were again conducted to ensure no violation of the assumptions of normality, linearity, multicollinearity and homoscedasticity. Age and gender were entered at step 1, explaining only 1% of the variance in experienced support from the clinic. After entering being taken seriously in step 2, the total variance explained by the model was 60.3%, *p* ≤ 0.0001.

#### 3.3.2. Family Self-Efficacy

Family self-efficacy was related to GF (r_s_ = 0.432), PhF (r_s_ = 0.440), RA (r_s_ = 0.416), RM (r_s_ = 0.431) and MF (r_s_ = 0.298). When adding the fatigue dimensions in a multiple regression model to assess their ability to predict the variation in family self-efficacy, only RM (Beta = 0.222, *p* = 0.004) contributed significantly to the variance in family self-efficacy. When proceeding with a hierarchical multiple regression to assess the ability of RM, to predict levels of family self-efficacy after controlling for the influence of parents’ gender and age, it was revealed that age and gender only explained 0.5% of the variance. After entering RM, the total variance explained by the model was 16%, *p* ≤ 0.0001.

## 4. Discussion

### Strengths and Limitations

This is the first study of all parents whose children are registered in the PECARE. The quality register provides a unique opportunity to study the whole population of the children’s parents, who are a prerequisite for the care. A strength of this study is the geographical deployment and coverage of Sweden as a whole.

The cohort is considered representative when compared to the children of non-responders.

This study used four instruments to cover different areas of parental concerns related to the research questions. However, there are few validated instruments designed to explore parents’ experiences and specifically intended for parents of children who are visually impaired. Castenada et al. have worked to develop questionnaires for measuring the quality of life of children with cataract and their parents [6], but more are needed for this group of patients and their parents.

A combination of both traditional and online questionnaires may have increased the response rate [30]. The children of non-responders had a mean age of 9 years—an age at which the visual system is fully developed and check-ups at the clinic are less frequent, which might have influenced the response rate. An additional factor that might have reduced the number of respondents in our study was that another study was conducted at the same time, partly on the same population, where 54.2% (*n* = 149) of the children of the non-responders were operated on at the clinic in which the other study took place, thus the parents might have been unwilling to become involved in a second study. In addition, only 20% of the respondents were fathers.

One limitation is that the questionnaires were in Swedish, which prevented non-Swedish speaking parents from participating. Only 8% of the parents were from a country other than Sweden, which might be reflected in the result. Finally, the family self-efficacy instrument has not been validated for Swedish use.

## 5. Reflections on the Findings

### 5.1. Fatigue

The main finding in this study was the prevalence of parental fatigue (38%), especially among those with children who had bilateral cataract. It is not unusual per se for new parents of even healthy children to suffer from fatigue, which has the potential to negatively influence parenting behaviors that are important for children’s well-being and development [31]. However, in light of the need for extensive parental caregiving to a child with cataract, it requires action on the part of healthcare professionals to adjust their information, support and demands, especially as fatigue is also considered to be a related but distinct construct from depression in the post-natal and early parenting period [32]. Co-morbidity increases parental burden and fatigue, which is confirmed by other studies [33,34]. If the child has additional disabilities, parents experience a lower level of family self-efficacy and less support from friends.

Fatigue also diminishes meaningfulness, which can have a negative effect on the willingness master necessary challenges, in this context handling contact lenses, eye drops and patching. Parents need to understand the reason for the required self-management activities. Because self-management and self-efficacy are significantly correlated, teaching strategies to promote self-efficacy may also promote self-management [35]. Is fatigue under reported? Here, the ophthalmological team needs to be present and strengthen the parents’ essential role as co-caregivers in the care of their child.

### 5.2. PREM and Being Taken Seriously

The strongest relationship identified in the study was between satisfaction with the care provided by the ophthalmological clinic and being taken seriously. Being taken seriously by the ophthalmological clinic explains over 60% of the variation in satisfaction with the care when controlled for parents’ age and gender. Studies indicate the importance of being taken seriously [36,37] and that being taken seriously is related to the perception of safe care [38]. When taken seriously, the person feels competent, which is a fundamental aspect of person-centered care [36]. Transferred to parents of children with cataract, this is crucial, as they have such an important role in the care of their child’s visual development. Measurement of parents’ self-efficacy, outcome expectations and task performance has the potential for application in both clinical and research settings, as a useful outcome measure for evaluating parent-focused interventions [39].

### 5.3. Gender Perspectives and Family Structure

The response rate of fathers in this study was 20%, thus their concerns need further investigation. Bearing that in mind, it is important to acknowledge gender differences. In this study, mothers were more fatigued than fathers. This might be correlated with the fact that mothers more often take parental leave, and therefore assume a greater responsibility for the care of their child, i.e., bringing the child to check-ups at the clinic and the daily routines with contact lenses and patching. Wilson concludes that new parents are vulnerable to fatigue and depressive symptoms, and there is a strong correlation between fatigue and depressive symptoms among women in the first two years after childbirth [40]. Pediatric cataract in Sweden is mostly diagnosed during the child’s first three months [41], a period that is challenging, even for parents of healthy children, to which one must add the shock of having to witness the baby undergo surgery to save her/his vision [4]. If the mother is the one on leave, it will be her responsibility to deal with eye drops, contact lenses and patching, as well as being alert to signs of complications such as infections and glaucoma. These factors might contribute to increasing fatigue.

There was also a gender difference in the sense of meaningfulness, as fathers expressed more difficulties than mothers. A previous study reported that fathers seemed to have a salutogenic approach that involves prioritizing health, goalsetting and progress [4]. This approach could be an asset, and needs to be introduced to both fathers and mothers by the ophthalmological team.

Apart from gender, another aspect that the ophthalmological team should address is family structure. Parents not living with each other experienced more difficulties managing the situation and also felt they received less support from the ophthalmological clinic.

Additionally, in this study, parents who had sons were more satisfied with their own efforts. The reason for this is unclear and requires further investigation.

### 5.4. Organizing Care to Support Parents

Effective self-management of a child’s chronic disease extends beyond typical parenting skills, to include symptom monitoring, treatment, medications, specialized nutrition, physical and psychosocial issues and financial planning [42]. Thus, the self-management load for the parents in this study is high—is self-management support equally high? While the ophthalmological team expects parents to assume this responsibility, it must also support and encourage them. This could be done by using a mobile application, facilitating communication, partnership and support in an easy and accessible way. Mobile applications for health have the potential to enable better self-management and can further improve the patients’ general self-efficacy and self-efficacy of activities that contribute to chronic disease management [43]. Likewise, when creating an individualized care plan for children in collaboration with the parents, it is essential to include the parents’ well-being. This is confirmed by Barbarian, who used PREM, where the parents emphasized the importance of creating an environment characterized by partnership and open communication [44].

These issues can be discussed and mastered within a solid and agreeable partnership with healthcare professionals.

This study has provided valuable information about the vulnerability of the parents of children with a cataract, which needs to be addressed by the ophthalmological team. While the team members cannot reduce the cause of parents’ fatigue, they can acknowledge it. Professionals need to develop and offer self-management support strategies to parents to help them cope despite fatigue. It would be interesting to explore whether novel therapies such as mindfulness and yoga could benefit this group and reduce their caregiver stress. In another population, Johansson et al. suggest that patients who suffer from mental fatigue after a stroke or traumatic brain injury may be helped by mindfulness-based stress reduction (MBSR) [45,46]. Likewise, it was concluded that yoga is a feasible intervention for, and is beneficial to, adolescents with cancer and their parents [47].

## 6. Conclusions

In conclusion, fatigue is significant among parents of children with cataract, especially those with bilateral cataract, and mothers are more burdened by fatigue than fathers. Mental fatigue and reduced motivation explain 45% of the variation in SOC, and are thus important aspects to consider. Being taken seriously is the key marker of satisfaction with care and the support from the professionals. The parents’ age and life situation affect how they perceive their own as well as the professionals’ effort and should be considered when tailoring family-centered care.

### 6.1. Clinical Implications

The ophthalmological team needs to:

- Affirm that parents of children with cataracts are tired and that mothers experience more fatigue than fathers.
- Acknowledge that parents of children with bilateral cataracts are more fatigued than other parents.
- Identify and pay extra attention to those parents who are not living with the other parent.

### 6.2. Future Research Questions

If the parents are tired, in what way does this affect the children?

Continue to ask the parents and children questions. PREM questions could be used nationally and administered through a digital app.

Explore gender differences among parents of children with cataracts.

## Figures and Tables

**Table 1:** Demographics of parents and children.

PARENTS	*N* = 231	*N*	%
Range: 27–65, Mean: 40.39, SD: 6.41, Median: 40			
Age	<40	106	45.9
	>40	124	53.7
Gender	mothers	185	80.1
	fathers	44	19.9
Education	high school	68	29.4
	university/college	161	69.7
Social status	living with other parent	201	87
	not living with other parent	29	12.6
Occupation	working/studying	201	87
	on parental leave	12	5.2
	on sick leave	3	0.9
Native country	Sweden	188	81.4
	other country	20	8.7
**CHILDREN**	***N* = 231**	***N***	**%**
**Age at study** (years)			
Range: 0–20, Mean: 7.6, SD: 3.89, Median: 7			
	0–6	97	42
	7–12	113	48.9
	13–20	21	9.1
**Age at surgery** (months)			
Range: 0–100, Mean: 23.45, SD: 27.44, Median: 8			
	0–3	99	42.9
	4–6	11	4.8
	7–12	11	4.8
	>12	110	47.6
gender	girls	111	48.1
	boys	120	51.9
laterality	unilateral	125	54.1
	bilateral	106	45.9
heredity	yes	42	18.2
	no	175	75.8
other disease	yes	27	11.7
	no	202	87.4
postoperative	yes	91	39.4
complications	no	140	60.6

**Table 2:** Scores from sense of coherence (SOC), Multidimensional Fatigue Inventory Scale (MFI-19), Multidimensional scale of perceived social support (MSPSS), Family self-efficacy and PREM-questions.

	*N*	Missing	Possible Range	Minimum	Maximum	Median	Percentiles	
SOC-Sense of Coherence							25	75
Total SOC	230	1	13–91	42	91	73	66	78
Comprehensibility	230	1	5–35	12	35	27	24	29
Manageability	230	1	4–28	11	28	22	19	24
Meaningfulness	230	1	4–28	12	28	24	22	26
MFI-19								
General Fatigue GF	231	0	5–24	4	20	11	8	15
Physical Fatigue PhF	231	0	5–24	3	20	8	5	12
Reduced Activity RA	231	0	5–24	3	20	8	5	11
Reduced Motivation RM	231	0	5–24	3	18	7	5	9
Mental Fatigue MF	231	0	5–24	2	14	6	4	9
MSPSS Social Support								
Significant Other	231	0	4–28	1	28	25	7	28
Family	231	0	4–28	1	28	23	7	28
Friends	231	0	4–28	1	28	19	6.75	27
Family Self-Efficacy	230	1	19–133	35	133	105	92.75	116
PREM								
Satisfaction with treatment from clinic	231	0	1–100	1	100	90	75	100
Satisfaction with own effort	230	1	1–100	30	100	80	70	95
Support from clinic	230	1	1–100	1	100	85	70	100
Taken seriously by clinic	231	0	1–100	1	100	95	80	100
